# LncRNA LINC01232 Enhances Proliferation, Angiogenesis, Migration and Invasion of Colon Adenocarcinoma Cells by Downregulating miR-181a-5p

**DOI:** 10.4014/jmb.2206.06032

**Published:** 2022-11-11

**Authors:** Yu Yuan, Zhou Long

**Affiliations:** 1 Department of Integrative Chinese and Western Medicine Anorectal Surgery, Yichun People’s Hospital, Yichun 336000, Jiangxi, P.R. China; 2 Deparment of Anorectal Surgery, Jingmen No.1 People’s Hospital, Diaodao District, Jingmen City, Hubei 448001, P.R. China

**Keywords:** LINC01232, colon adenocarcinoma, miR-181a-5p, proliferation, migration, invasion, angiogenesis

## Abstract

LncRNAs play crucial roles in the progression of colon adenocarcinoma (COAD), but the role of LINC01232 in COAD has not received much attention. The present study was designed to explore the related mechanisms of LINC01232 in the progression of COAD. LINC01232, miR-181a-5p, p53, c-myc, Bcl-2, cyclin D1, p16, Bax, VEGF, E-cadherin, vimentin, N-cadherin and SDAD1 expressions were determined by western blot and qRT-PCR. CCK-8, tubule formation, and Transwell assays were employed to detect proliferation, angiogenesis, and migration/invasion of COAD cells, respectively. The relationship between LINC01232 and miR-181a-5p was predicted by LncBase Predicted v.2, and then verified through dual luciferase reporter gene assay. According to the results, LINC01232 was highly expressed in COAD cells and enhanced proliferation, angiogenesis, migration, and invasion of COAD cells. Downregulated LINC01232 promoted expression of p53 and p16, and inhibited c-myc, Bcl-2 and cyclin D1 expressions in COAD cells, while upregulation of LINC01232 generated the opposite effects. LINC01232 was negatively correlated with miR-181a-5p while downregulated miR-181a-5p could reverse the effects of siLINC01232 on cell proliferation, angiogenesis, migration, and invasion. Similarly, miR-181a-5p mimic could also offset the effect of LINC01232 overexpression. SiLINC01232 increased the expressions of Bax and E-cadherin, and decreased the expressions of VEGF, vimentin, N-cadherin and SDAD1, which were partially attenuated by miR-181a-5p inhibitor. Collectively, LINC01232 enhances the proliferation, migration, invasion, and angiogenesis of COAD cells by decreasing miR-181a-5p expression.

## Introduction

Colon adenocarcinoma (COAD) is a familiar pernicious neoplasm that occurs in the colon mucosa, mostly harming middle-aged and elderly people aged 30-69 years, with obvious gender difference (males are more susceptible than females) [1, 2]. According to global cancer statistics in 2018, there were 1.1 million new cases of COAD (accounting for 6.1% of all new cancer cases worldwide), with more than 550,000 deaths (accounting for 5.8% of all cancer deaths worldwide) [2]. Despite improvements in treatment in recent years, the five-year survival rate for COAD is still only 50-60% [3]. Due to the insidious early symptoms of COAD, a great number of patients are diagnosed in the moderate-to-advanced stages, and have already developed invasion and metastasis, leading to the loss of optimal treatment time [4]. In addition, 30-50% of patients with COAD sustain recurrence and metastasis after surgery [5, 6]. Therefore, it is of great significance to explore the mechanism of tumor cell metastasis in the treatment of COAD.

Long non-coding RNA (LncRNA) is a kind of non-coding RNA that is longer than 200 nucleotides [7]. LncRNAs do not code functional proteins, but are involved in the formation of a complex and critical network of gene expression regulation to subtly modulate gene expression [8]. Recent studies have shown that lncRNA regulates the expressions of oncogenes and tumor suppressor genes at the epigenetic and transcriptional translation levels, participates in biological processes such as cell proliferation, migration and invasion, and affects the occurrence and development of tumors [9-11]. LINC01232 is a newly identified lncRNA which has not received extensive attention at present. Li *et al*. proposed that LINC01232 exerts its carcinogenic activities in pancreatic cancer by regulating TM9SF2 in pancreatic adenocarcinoma progression [12]. Similarly, Meng *et al*. demonstrated that LINC01232 is a promising target molecule for pancreatic cancer treatment [13]. However, the role of LINC01232 in COAD is unclear.

One way in which lncRNAs participate in cellular biological processes is through competitive binding to target micro (mi)RNAs, thereby affecting the expressions of downstream genes [14, 15]. MiRNAs are also a kind of non-coding RNA, and have aroused considerable concern among researchers in recent years. According to a previous study, LINC01232 could competitively bind to miR-654-3p, and reduce its expression in ESCC cells, thus promoting the expression of HDGF [16]. Nevertheless, the regulation of miRNA by LINC01232 in colon cancer is still undefined. Through bioinformatics analysis, we found that LINC01232 could bind to miR-181a-5p. It is noteworthy that the involvement of miR-181a-5p in the progression of COAD has been confirmed [17, 18]. On this basis, we hypothesized that LINC01232 affects the progression of COAD by modulating miR-181a-5p.

In the present study, we first measured the expressions of LINC01232 and miR-181a-5p in COAD and normal cells. The roles of LINC01232 and miR-181a-5p in the progression of COAD were then determined by regulating their expressions. Therefore, our study may provide a significant therapeutic target for the treatment of COAD.

## Materials and Methods

### Cell Culture

Normal colon fibroblasts (CCD-18Co) and COAD cells (LS174T, LOVO, HT29, HCT116 and SW-620) were obtained from ATCC (USA). CCD-18Co and LS174T cells were maintained in DMEM medium (12491-015, Gibco, USA). LOVO cells were grown in F-12K medium (30-2004, ATCC). HCT116 and HT-29 cells were cultured in McCoy's 5a medium (30-2007, ATCC). SW-620 cells were incubated in Leibovitz's L-15 medium (30-2008, ATCC). All media were supplemented with 10% fetal bovine serum (FBS, 10099-141, Gibco) and 1%Penicillin-Streptomycin (15070063, Gibco). CCD-18Co, LS174T, LOVO, HT29 and HCT116 cells were cultured in an incubator at 37°C with 5% CO_2_, while SW-620 cells were cultivated in the incubator at 37°C without CO_2_.

### Cell Transfection

SW-620 or LOVO cells were placed in a 6-well plate with 3 × 10^5^ cells per well. When the cell confluence reached 70-80%, small interfering RNA for LINC01232 (siLINC01232, siG180418054136-1-5, RIBOBIO, China), LINC01232 overexpression plasmid, miR-181a-5p mimic (miR10000256-1-5, RIBOBIO), miR-181a-5p inhibitor (miR20000256-1-5, RIBOBIO) and their corresponding controls (NC, pcDNA3.1 empty vector; siNC: siN0000002-1-10; mimic control (MC): miR1N0000001-1-10; inhibitor control (IC): miR2N0000001-1-10; RIBOBIO) were transfected into SW-620 or LOVO cells. LINC01232 overexpression plasmid and its negative control were constructed by GenePharma (China). Briefly, 4 μg siLINC01232, LINC01232 overexpression plasmid or the controls and 50 nM miR-181a-5p mimic, miR-181a-5p inhibitor or the controls were diluted with 250 μl Opti-MEM (31985-070, Gibco). Ten microliters of Lipofection 2000 (11668-027, Invitrogen, USA) was also diluted with 250 μl Opti-MEM. Then, plasmids or oligonucleotide and Lipofection 2000 were mixed for 20 min. Afterwards, the mixture was added into the corresponding cells. The medium containing the transfection reagent was changed to the normal medium after 6 h, and the transfection efficiency was identified by western blot or quantitative reverse transcription polymerase chain reaction (qRT-PCR) after 24 h.

### qRT-PCR

Total RNA of cells was extracted by Trizol reagent (15596-018, Invitrogen), the concentration of which was determined by a microplate reader (Molecular Devices, USA). Next, 1 μg RNA was reversely transcribed into cDNA, which was used for qPCR, with a First cDNA Synthesis Kit (RR037A, TaKaRa, Japan). In short, 20 μl reaction solution was prepared as follows: 2 μl cDNA, 10 μl SYBR Mix (RR820A, TaKaRa), 0.8 μl forward primer, 0.8 μl reverse primer and 6.4 μl sterile water. Then, the mixture was amplified under the following reaction conditions: 95°C for 30 s, 40 cycles of 95°C for 3 s, and 60°C for 30 s. The primers were obtained from Sangon (China) and RIBIBIO, and the sequences were listed in Table 1. U6 and β-actin were internal references. Finally, the CT value obtained from a 7900 Real-Time PCR System (Biosystems, USA) was calculated by 2^-ΔΔCT^ method [19].

### Cell Counting Kit (CCK)-8

After transfection for 24 h, SW-620 and LOVO cells were inoculated in a 96-well plate (3000/well). Cell culture was continued for another 24 and 48 h, and the CCK-8 assay was carried out. Then, 10 μl CCK-8 reagent (C0038, Beyotime, China) was added to each well, mixed with cells gently, and placed in the incubator for 3 h. At the end of incubation, the culture plate was taken out and placed in a microplate reader to detect the light absorption at 450 nm.

### Tubule Formation Assay

Fifty microliters of matrix glue (356234, Becton, Dickinson and Company, USA) was added to a 6-well plate, which was then incubated for 30 min. After transfection for 24 h, SW-620 and LOVO cells were collected and resuspended in 2 ml medium. Then, 50 μl cells were added to the pre-coagulated matrix glue for further culture in the incubator. After 12 h, the culture plate was photographed under an inverted microscope (POMEAS, China), and Image J (1.8.0, National Institutes of Health, Germany) was used to calculate the length of the tubule.

### Transwell Assay

Transfected or untransfected cells were resuspended in 2 ml medium without FBS. For invasion detection, the upper chamber of the Transwell plate (3428, Corning, USA) was first uniformly coated with the matrigel (Corning) and placed in the incubator for 4 h. Then, 100 μl pre-prepared cells were put into the upper chamber, and 750 μl medium containing 10% FBS was put into the lower chamber. Following culture for 48 h, the cells of the upper chamber were gently removed with a cotton swab. Cells invading the lower chamber were immersed in 4%paraformaldehyde (P0099, Beyotime) for 30 min, and then stained by crystal violet (C0121, Beyotime) for 30 min. Next, the invading cells were photographed with an inverted microscope, and 5 fields were randomly selected to count the number of cells. The Transwell assay for migration detection was the same as above except that matrigel was not required.

### Dual-Luciferase Reporter Gene Assay

Potential 3’UTR-specific binding sites of LINC01232 for miR-181a-5p were predicted by LncBase Predicted v.2 (http://diana.imis.athena-innovation.gr/DianaTools/index.php?r=site/page&view=software), and the 3’UTR sequence of LINC01232 was obtained from the NCBI database (https://www.ncbi.nlm.nih.gov/). Then, wild-type or mutant-type LINC01232-3’UTR was inserted into pmiRGLO vector (E1303, Promega, USA) to acquire LINC01232-WT and LINC01232-MUT pmiRGLO plasmids.

SW-620 or LOVO cells were seeded in a 6-well plate (3 × 105/well). When the cell confluence reached 70-80%, LINC01232-WT or LINC01232-MUT plasmid and miR-181a-5p mimic or mimic control were co-transfected into SW-620 or LOVO cells. The luciferase activity was measured by using a dual-luciferase reporter gene detection system (E1910, Promega) 48 h after transfection.

### Western Blot

Total protein of cells was extracted by RIPA lysis (P0013B, Beyotime) supplemented with 1% protease inhibitor (P1030, Beyotime). The protein extract was centrifuged in a 4°C centrifuge at 1200 ×*g* for 10 min, and then the supernatant was transferred to a new EP tube. The concentration of the protein extract was determined using the BCA kit (P0012, Beyotime). Then, 30 μg protein samples and 4 μl marker (PR1910, Solarbio, China) were added into a hole of SDS-PAGE gel (P0012A, Beyotime) for 2 h of electrophoresis. The proteins isolated by SDS-PAGE gel were then transferred to a PVDF membrane (ISEQ00010/IPVH00010, Millipore, USA), and sealed at room temperature with 5% skim milk (D8340, Solarbio) for 1 h. Later, the membrane was incubated with primary antibodies (p53, ab26, 1:1000, mouse, Abcam, Cambridge, MA, USA; Bcl-2, ab182858, 1:2000, rabbit, Abcam; Bax, ab32503, 1:5000, rabbit, Abcam; VEGF, AV202, 1:1000, rabbit, Beyotime; E-cadherin, ab238099, 1:1000, mouse, Abcam; Vimentin, ab92547, 1:5000, rabbit, Abcam; N-cadherin, ab76011, 1:5000, rabbit, Abcam; SDAD1, sc-515320, 1:1000, mouse, Santa Cruz Biotechnology at 4°C overnight. The next day, the membrane was cultured with secondary antibodies (Goat Anti-Rabbit IgG H&L: ab6721, 1:10000, abcam, USA; Goat Anti-Mouse IgG H&L: ab6789, 1:5000, abcam) at room temperature for 1 h. Then, the membrane was visualized by using ECL chemiluminescence liquid (WBKLS0500, Millipore) and exposed to a specific instrument (Bio-Rad, USA). The gray values of the protein bands were analyzed by Image J.

### Statistical Analysis

Data were expressed as the means ± SD. Differences among groups were determined statistically using analysis of variance (ANOVA). Statistical analyses were performed by the SPSS software (19.0, IBM, USA). *p* < 0.05 indicated statistically significant difference.

## Results

### LINC01232 Was Highly Expressed in COAD Cells

**Fig. 1:**
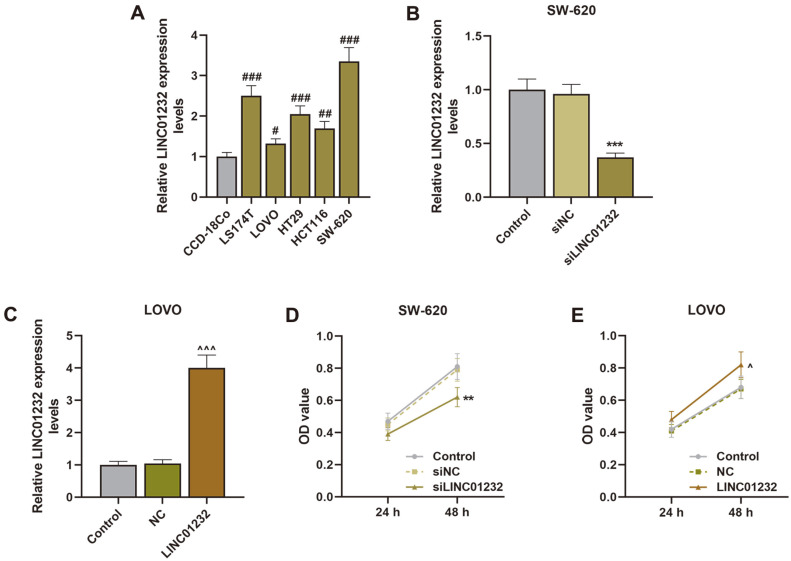
LINC01232 was highly expressed in COAD cells, and enhanced cell proliferation. (**A**). The expression of LINC01232 in cells was determined by qRT-PCR. β-Actin served as an internal reference. (**B-C**). The transfection efficiency of siLINC01232 and LINC01232 overexpression plasmid was detected by qRT-PCR. β-Actin served as an internal reference. (**D-E**). CCK-8 assay was used to measure the proliferation of SW-620 and LOVO cells transfected or untransfected with siLINC01232 and LINC01232 overexpression plasmid. #vs. CCD-18Co, *vs. siNC, ^vs. NC; ^#^ *p* < 0.05, ^##^ *p* < 0.01, ^###^or^***^or^^^ *p* < 0.001. COAD, colon adenocarcinoma; qRT-PCR, quantitative reverse transcription polymerase chain reaction; CCK-8, cell counting kit-8.

For the first time, we identified the expression of LINC01232 in COAD cells. According to Fig. 1A, the qRT-PCR detection results showed the expression of LINC01232 in COAD cells to be higher than that in normal colon fibroblasts (*p* < 0.05, *p* < 0.01, *p* < 0.001). As the expression of LINC01232 was the highest in SW-620 cells and the lowest in LOVO cells, the two cells were selected as subjects for further experiments. In addition, due to the high expression level of LINC01232 in SW-620 cells, we transfected siLINC01232 into SW-620 cells to downregulate the level of LINC01232, and thus unveil its role (Fig. 1B, *p* < 0.001). By contrast, we then upregulated LINC01232 expression in LOVO cells through transfection of LINC01232 overexpression plasmid, and the transfection efficiency was exhibited in Fig. 1C (*p* < 0.001).

### LINC01232 Overexpression Promoted Cell Proliferation, Migration, Invasion, and Angiogenesis

**Fig. 2:**
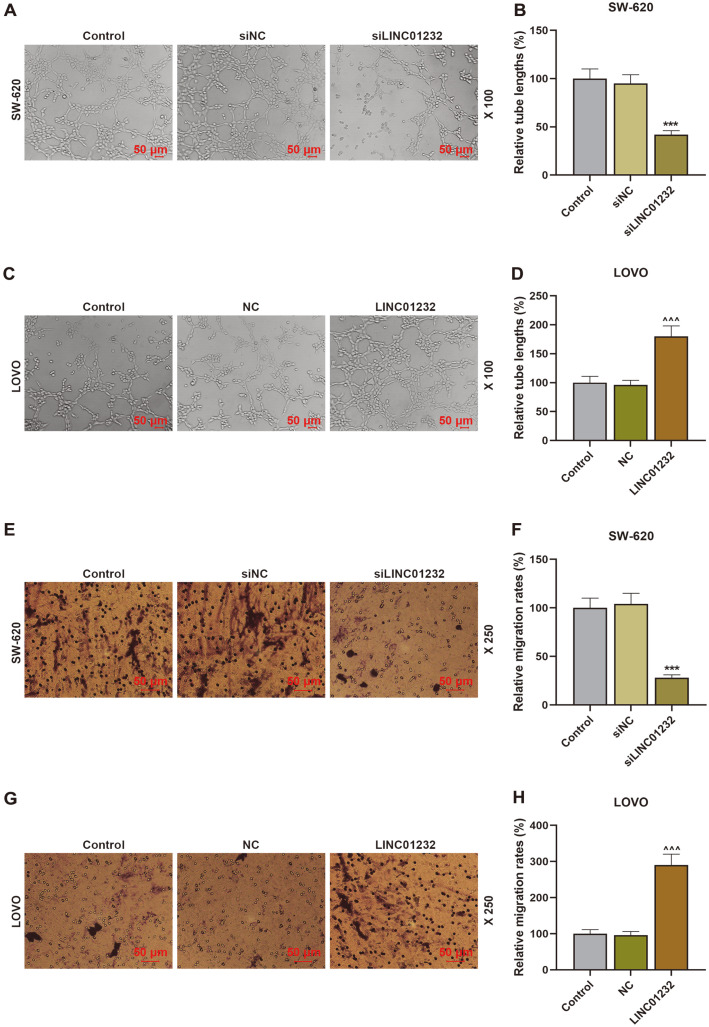
LINC01232 overexpression enhanced angiogenesis and migration of COAD cells. (**A-D**). Tubule formation assay was applied to determine the angiogenesis of SW-620 and LOVO cells transfected or untransfected with siLINC01232 and LINC01232 overexpression plasmid. (**E-H**). Transwell assay was employed to detect the migration of SW- 620 and LOVO cells transfected or untransfected with siLINC01232 and LINC01232 overexpression plasmid. *vs. siNC, ^vs. NC; ***or^^^ *p* < 0.001. COAD, colon adenocarcinoma.

**Fig. 3:**
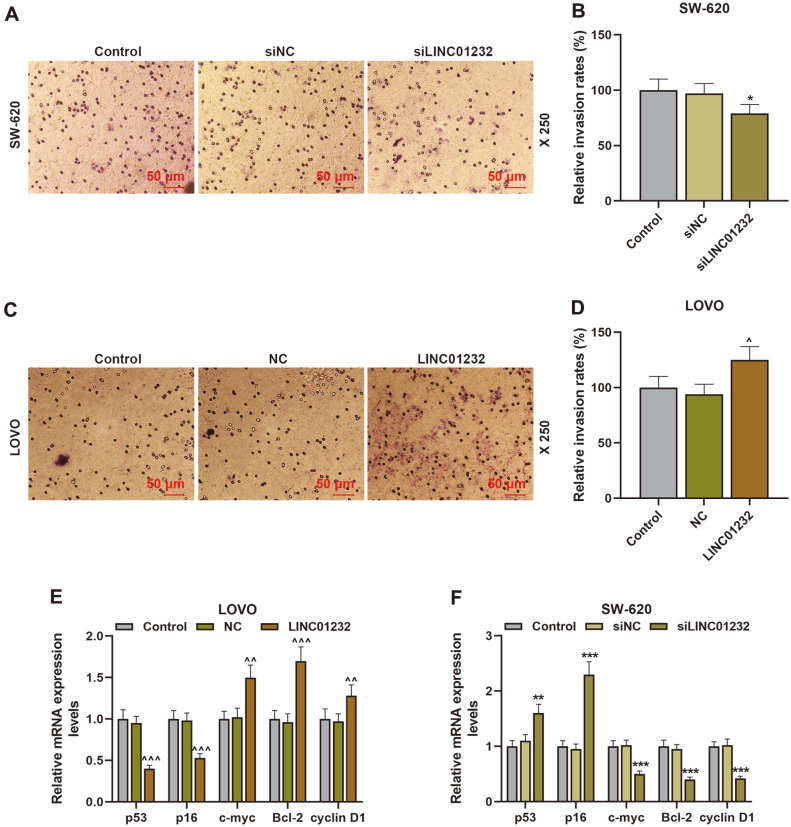
LINC01232 overexpression enhanced cell invasion and expressions of c-myc, Bcl-2, and cyclin D1, and inhibited p53 and p16 expressions. (**A-D**). Transwell assay was conducted to test the invasion of SW-620 and LOVO cells transfected or untransfected with siLINC01232 and LINC01232 overexpression plasmid. (**E-F**). The expressions of p53, p16, c-myc, Bcl-2 and cyclin D1 were detected by qRT-PCR. β-Actin served as an internal reference. *vs. siNC, ^vs. NC; *or ^ *p* < 0.05, **or ^^ *p* < 0.01, ***or^^^ *p* < 0.001. c-myc, cellular-myelocytomatos; Bcl-2, B-cell lymphoma-2; p53, protein 53; p16, multiple tumor suppressor 1; qRT-PCR, quantitative reverse transcription polymerase chain reaction.

Next, the effects of LINC01232 on COAD cell proliferation, migration, invasion, and angiogenesis were identified by CCK-8, Transwell, and tubule formation assays. The assay results indicated that LINC01232 silencing suppressed the proliferation, migration, invasion, and angiogenesis of SW-620 cells, while LINC01232 overexpression enhanced the proliferation, migration, invasion, and angiogenesis of LOVO cells, in comparison to the control (Figs. 1D-1E, 2A-2H, 3A-3D, *p* < 0.05, *p* < 0.01, *p* < 0.001). Then, the expressions of p53, p16, c-myc, Bcl-2 and cyclin D1 were also quantified. As detailed in Figs. 3E-3F, the upregulated LINC01232 dwindled p53 and p16 expressions and elevated c-myc, Bcl-2 and cyclin D1 expressions in LOVO cells, while the downregulated LINC01232 augmented p53 and p16 expressions and diminished c-myc, Bcl-2, and cyclin D1 expressions in SW-620 cells (*p* < 0.01, *p* < 0.001).

### LINC01232 Was Negatively Correlated with miR-181a-5p

**Fig. 4:**
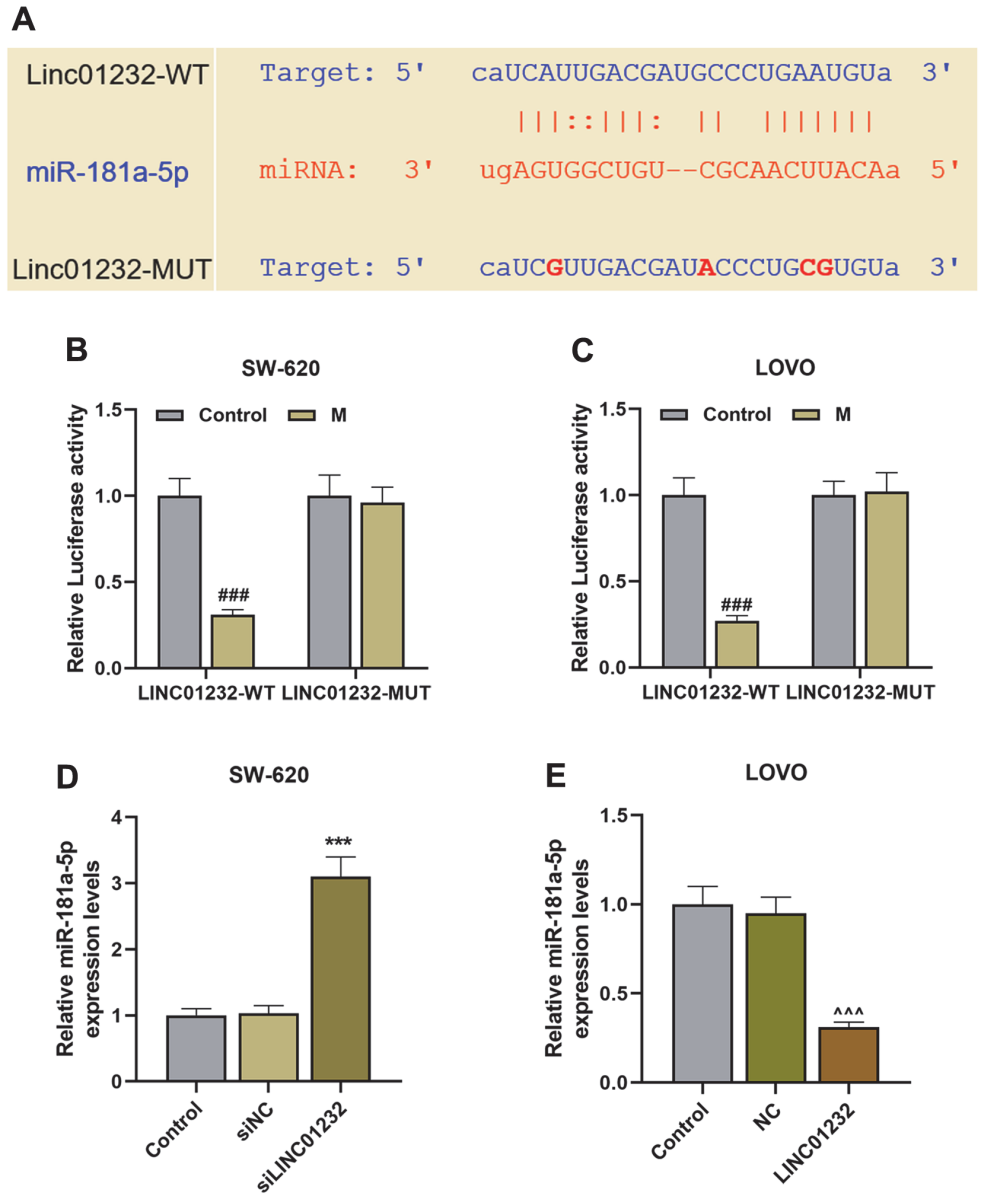
LINC01232 was negatively correlated with miR-181a-5p. (**A**). The binding site of LINC01232 and miR-181a- 5p was predicted by LncBase Predicted v.2. (**B-C**). The relationship of LINC01232 and miR-181a-5p was verified by dualluciferase reporter gene assay. (**D-E**). The expression of miR-181a-5p in SW-620 and LOVO cells transfected or untransfected with siLINC01232 and LINC01232 overexpression plasmid was determined by qRT-PCR. U6 served as an internal reference. ^#^vs. Control, *vs. siNC, ^vs. NC; ^***^or ^###^or ^^^ *p* < 0.001. COAD, colon adenocarcinoma; qRT-PCR, quantitative reverse transcription polymerase chain reaction.

LncBase Predicted v.2 was employed to predict the binding site of LINC01232 and miR-181a-5p, and the LINC01232 sequence of the binding site mutation was designed (Fig. 4A). Notably, the prediction result was verified by dual-luciferase reporter gene assay. Co-transfection of LINC01232-WT and miR-181a-5p mimic could reduce the luciferase activity of cells, while co-transfection of LINC01232-MUT and miR-181a-5p mimic had no significant effect on the luciferase activity of cells, as compared with their control (Figs. 4B and 4C). Subsequently, we found that downregulation of LINC01232 enhanced, but upregulation of LINC01232 inhibited the expression of miR-181a-5p, when compared with their control (Figs. 4D and 4E, *p* < 0.001).

### LINC01232 Overexpression Facilitated Cell Proliferation, Migration, Invasion, and Angiogenesis by Inhibiting miR-181a-5p Expression

**Fig. 5:**
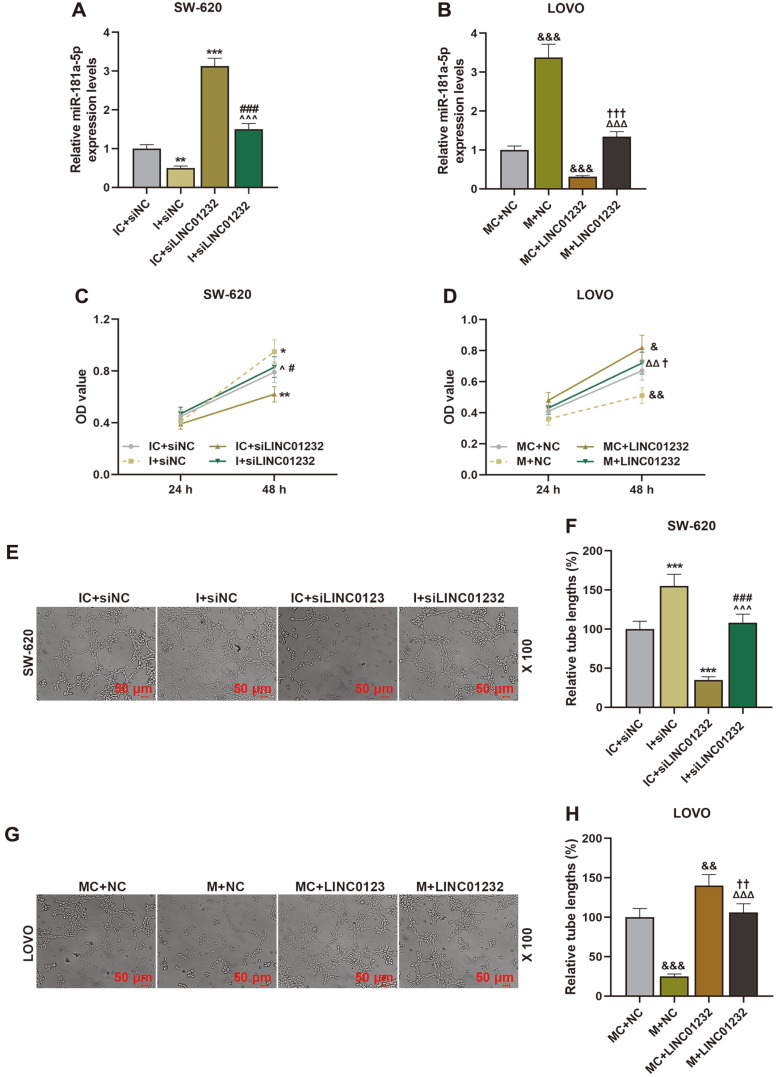
LINC01232 overexpression facilitated cell proliferation and angiogenesis by decreasing miR-181a-5p level. (**A-B**). The transfection efficiency of miR-181a-5p mimic and miR-181a-5p inhibitor was detected by qRT-PCR. U6 acted as an internal reference. (**C-D**). CCK-8 assay was carried out to examine the proliferation of SW-620 and LOVO cells transfected or untransfected with siLINC01232, LINC01232 overexpression plasmid, miR-181a-5p mimic and miR-181a-5p inhibitor. (**E-H**). Tubule formation assay was applied to determine the angiogenesis of SW-620 and LOVO cells transfected or untransfected with siLINC01232, LINC01232 overexpression plasmid, miR-181a-5p mimic and miR-181a-5p inhibitor. ^*^vs. IC+siNC, ^vs. I+siNC, ^#^vs. IC+siLINC01232, ^&^vs. MC+NC, ^Δ^ vs. M+NC, ^†^vs. MC+LINC01232; ^*^or^or^#^or^&^or^†^ *p* < 0.05, **or ^ΔΔ^ or^††^ or^&&^ *p* < 0.01, ^***^or^^^ or ^###^ or^&&&^ or ^ΔΔΔ^ or ^†††^*p* < 0.001. qRT-PCR, quantitative reverse transcription polymerase chain reaction; CCK-8, cell counting kit-8; I, miR-181a-5p inhibitor; IC, inhibitor control; M, miR-181a-5p mimic; MC, mimic control; siNC, siRNA negative control.

**Fig. 6:**
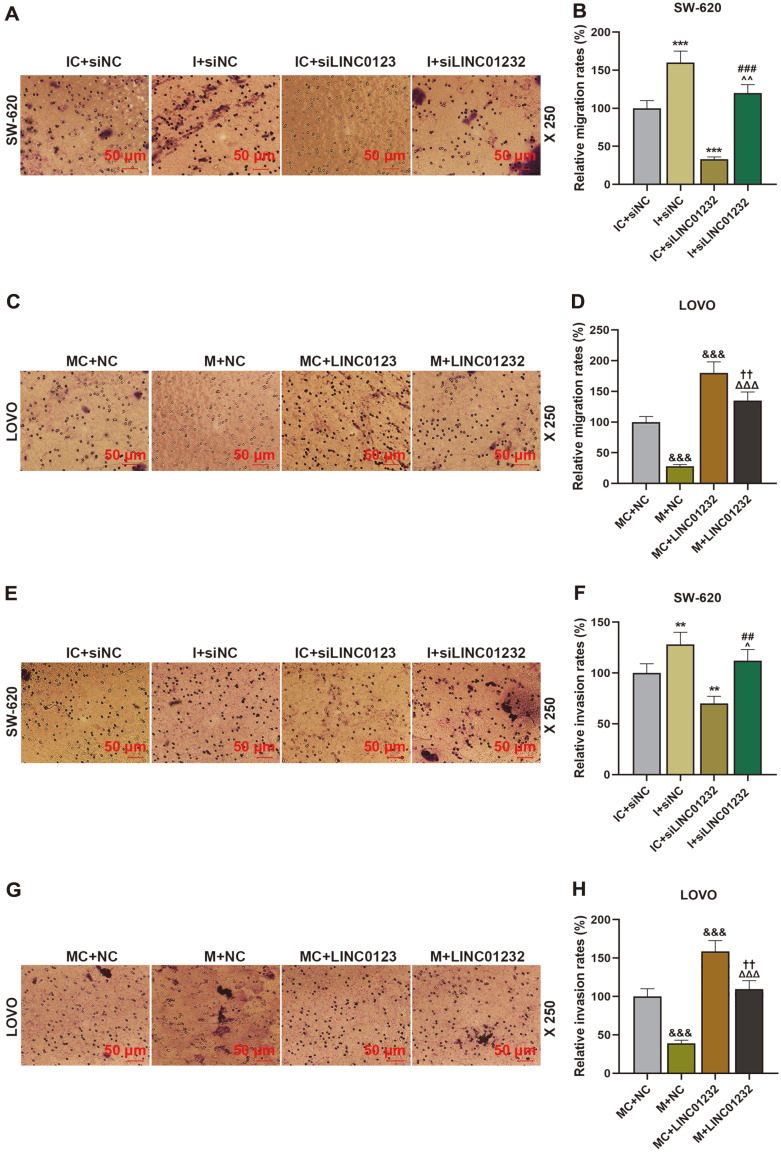
LINC01232 overexpression enhanced cell migration and invasion by decreasing miR-181a-5p level. (**A-H**). Transwell assay was performed to test the migration and invasion of SW-620 and LOVO cells transfected or untransfected with siLINC01232, LINC01232 overexpression plasmid, miR-181a-5p mimic and miR-181a-5p inhibitor. ^*^vs. IC+siNC, ^vs. I+siNC, ^#^vs. IC+siLINC01232, ^&^vs. MC+NC, ^Δ^ vs. M+NC, ^†^vs. MC+LINC01232; ^ *p* < 0.05, ^^or^**^or ^##^ or^††^ *p* < 0.01, ^***^or ^###^ or^&&&^ or ^ΔΔΔ^ *p* < 0.001. I, miR-181a-5p inhibitor; IC, inhibitor control; M, miR-181a-5p mimic; MC, mimic control; siNC, siRNA negative control.

To further investigate the effects of LINC01232 and miR-181a-5p on the progression of COAD, siLINC01232, LINC01232 overexpression plasmid, miR-181a-5p mimic and miR-181a-5p inhibitor were transfected to COAD cells. The transfection efficiency of miR-181a-5p mimic and miR-181a-5p inhibitor was depicted in Figs. 5A-5B. MiR-181a-5p inhibitor significantly decreased miR-181a-5p expression in SW-60 cells of the I+siNC group, compared with that of the IC+siNC group; and miR-181a-5p mimic increased miR-181a-5p expression in LOVO cells of M+NC group relative to that of MC+NC group (*p* < 0.01, *p* < 0.001). LINC01232 overexpression inhibited, while LINC01232 silencing promoted, the expression of miR-181a-5p; moreover, upregulation or downregulation of LINC01232 partially offset the regulatory effect of miR-181a-5p mimic or miR-181a-5p inhibitor on miR-181a-5p expression (Figs. 5A and 5B, *p* < 0.001). Afterwards, CCK-8, Transwell and tubule formation assays proved that miR-181a-5p inhibitor enhanced the proliferation, migration, invasion, and angiogenesis of SW-60 cells, and in contrast, miR-181a-5p mimic impeded the proliferation, migration, invasion, and angiogenesis of LOVO cells, as contrasted with their control (Figs. 5C-5H, 6A-6H, *p* < 0.05, *p* < 0.01, *p* < 0.001). Similarly, miR-181a-5p mimic or miR-181a-5p inhibitor could partially reverse the effects of LINC01232 overexpression or silencing on proliferation, migration, invasion, and angiogenesis of COAD cells (Figs. 5C-5H, 6A-6H, *p* < 0.05, *p* < 0.01, *p* < 0.001).

### LINC01232 Overexpression Increased Bcl-2, VEGF, Vimentin, N-Cadherin and SDAD1 Expressions and Decreased p53, Bax and E-Cadherin Expressions by Inhibiting miR-181a-5p Expression.

**Fig. 7:**
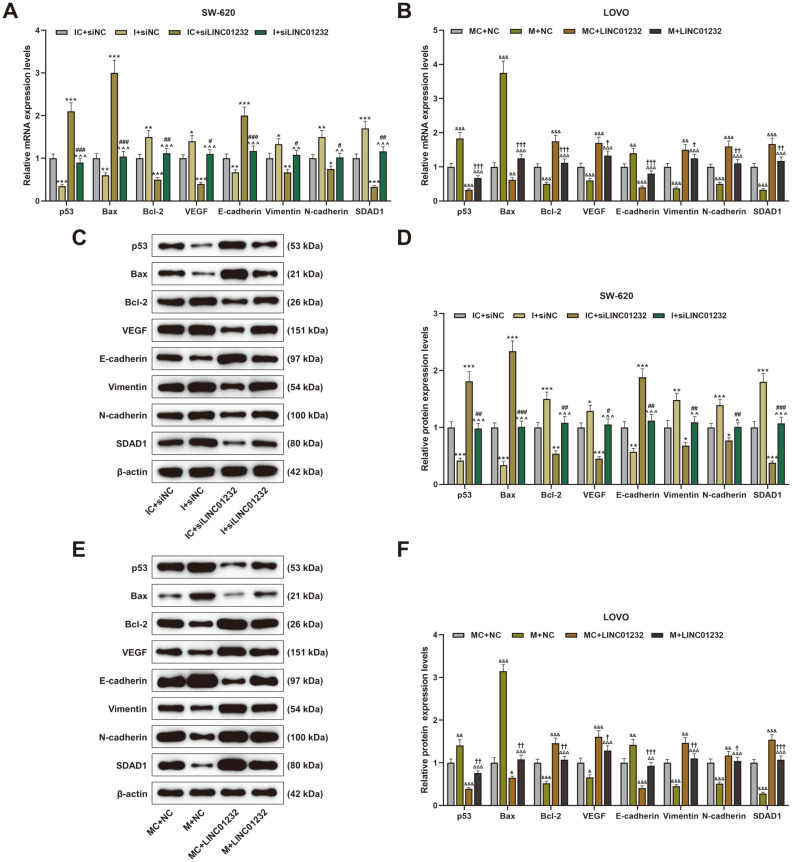
LINC01232 overexpression increased Bcl-2, VEGF, vimentin, N-cadherin and SDAD1 expressions and decreased p53, Bax and E-cadherin expressions by lessening miR-181a-5p level. (**A-F**). The expressions of p53, Bcl-2, Bax, VEGF, vimentin, E-cadherin, N-cadherin and SDAD1 in SW-620 and LOVO cells transfected or untransfected with siLINC01232, LINC01232 overexpression plasmid, miR-181a-5p mimic and miR-181a-5p inhibitor were determined by qRT-PCR or western blot. β-Actin served as an internal reference. ^*^vs. IC+siNC, ^vs. I+siNC, ^#^vs. IC+siLINC01232, ^&^vs. MC+NC, ^Δ^ vs. M+NC, ^†^vs. MC+LINC01232; ^*^ or ^#^ or^&^ or ^†^ *p* < 0.05, ^**^or^^ or ^##^ or^&&^ or ^ΔΔ^ or ^††^ *p* < 0.01, ^***^or^^^ or ^###^ or^&&&^ or ^ΔΔΔ^ or ^†††^*p* < 0.001. p53, protein 53; Bcl-2, B-cell lymphoma-2; VEGF, vascular endothelial growth factor; SDAD1, SDA1 domain containing 1; Bax, Bcl-2-associated X; I, miR-181a-5p inhibitor; IC, inhibitor control; M, miR-181a-5p mimic; MC, mimic control; siNC, siRNA negative control.

To further verify the above results, the expressions of Bcl-2, VEGF, vimentin, N-cadherin, SDAD1, p53, Bax and E-cadherin in COAD cells were detected. The results reflected that miR-181a-5p inhibitor decreased p53, Bax, and E-cadherin expressions, and increased Bcl-2, VEGF, vimentin, N-cadherin, and SDAD1 expressions in SW-620 cells in comparison to the control, and LINC01232 knockdown exerted opposite effects (Figs. 7A, 7C, 7D, *p* < 0.05, *p* < 0.01, *p* < 0.001). Interestingly, the regulatory effect of LINC01232 silencing on the above proteins was partially neutralized by miR-181a-5p inhibitor (Figs. 7A, 7C, 7D, *p* < 0.05, *p* < 0.01, *p* < 0.001). By contrast, miR-181a-5p mimic enhanced p53, Bax, and E-cadherin expressions and suppressed Bcl-2, VEGF, vimentin, N-cadherin, and SDAD1 expressions in LOVO cells, relative to the control, and LINC01232 overexpression generated inverse effects (Figs. 7B, 7E, 7F, *p* < 0.05, *p* < 0.01, *p* < 0.001). The regulatory effect of LINC01232 overexpression on these proteins was also partially offset by miR-181a-5p mimic (Figs. 7B, 7E, 7F, *p* < 0.05, *p* < 0.01, *p* < 0.001).

## Discussion

Colon adenocarcinoma is one of the most common cancers of the digestive tract globally, and lncRNAs have been evidenced to play vital roles in colon carcinogenesis and progression [20]. In this study, we found that the expression of LINC01232 was different in various cell lines, and abnormally high in COAD cells. We believed that this difference may stem from the different origins of cell lines, and the different phenotypes of each cell, which enable them to create the appropriate microenvironment [21]. Metastasis and invasion are the leading causes of death in COAD patients [22]. Here, we explored the relationship between LINC01232 and COAD cell migration and invasion by silencing or overexpressing LINC01232. From the results of Transwell assay, we could conclude that LINC01232 silencing hindered cell migration and invasion, and that LINC01232 overexpression had opposite effects. This suggested that LINC01232 was indeed involved in the migration and invasion of COAD cells. In addition, we also unraveled that LINC01232 overexpression enhanced the proliferation of COAD cells. Notably, abnormal cell proliferation is the basis of tumorigenesis [23]. Thus, LINC01232 has been confirmed to be implicated in the progression of colon cancer in our study.

To further verify the results of the above experiments, the expressions of p53, p16, c-myc, Bcl-2, and cyclin D1 were quantitated. P53 is a tumor suppressor gene that is mutated in about 50% of malignant tumors [24]. Thus, p53 could be used as a predictor of the progression from precancerous lesions to true malignant tumors [25]. Besides, an earlier study also proved that the expression of p53 was closely related to the invasion and lymphatic metastasis of COAD [26]. Likewise, p16 gene is also a critical tumor suppressor gene, which will lead to the proliferation of malignant cells after inactivation [27]. In line with a recent study, abnormality in the cyclinD1-CDK-p16-pRb pathway is the genetic basis of tumor development [28]. Overexpression of cyclin D1 protein will enhance the binding of cyclin D1 to cyclin-dependent kinases, further stimulate cell division, promote excessive cell proliferation, inhibit cell apoptosis, and finally lead to carcinogenesis [29]. Furthermore, as a nuclear transcription factor, c-myc could promote cell proliferation, enable cells at rest to enter into proliferation, and transform cells into undergoing malignant changes [30]. Predictably, we examined the expression of Bcl-2, a well-known apoptotic inhibitor [31]. In light of the data, overexpression of LINC01232 could decrease p53 and p16 expressions, and increase c-myc, Bcl-2 and cyclin D1 expressions. These results further confirmed that LINC01232 is involved in the proliferation, migration, and invasion of COAD cells. Furthermore, we noted that LINC01232 affected angiogenesis in colon cancer cells. It is worth mentioning that angiogenesis is vital for the rapid growth and metastasis of solid tumors [32]. Our results suggested that LINC01232 silencing attenuated, but LINC01232 overexpression strengthened, the tubule formation of COAD cells. The angiogenesis promoted by LINC01232 creates a strong condition for the development of COAD.

MiR-181a-5p, a targeted binding molecule downstream gene of LINC01232, has been demonstrated to be associated with the progression of COAD [17, 18]. The targeting relationship between miR-181a-5p and LINC01232 has also been verified in our study. We also corroborated that miR-181a-5p was dramatically lowly expressed in COAD [18, 33]. To further explore the roles of miR-181a-5p and LINC01232 in the progression of COAD, we simultaneously knocked down or overexpressed miR-181a-5p and LINC01232 in COAD cells. As previously mentioned, LINC01232 knockdown would dampen COAD cell proliferation, migration, invasion, and angiogenesis. Expectedly, downregulation of miR-181a-5p boosted proliferation, migration, invasion, and angiogenesis of COAD cells. In addition, downregulation/upregulation of miR-181a-5p could attenuate the effects of LINC01232 silencing/overexpression on the proliferation, migration, invasion, and angiogenesis of COAD cells. This implied that LINC01232 could enhance the proliferation, migration, invasion, and angiogenesis of COAD cells by inhibiting miR-181a-5p expression.

Then, we continued to verify the above results by detecting proliferation-, migration-, invasion-, and angiogenesis-related proteins. In addition to p53 and Bcl-2 expressions, Bax, VEGF, E-cadherin, vimentin, N-cadherin and SDAD1 expressions were also detected. Bax is a well-known pro-apoptotic protein in contrast to Bcl-2, an anti-apoptotic protein [34]. Particularly, VEGF is a functional glycoprotein with high biological activity [35]. It is the only growth factor specifically acting on vascular endothelial cells, and is most directly involved in inducing tumor angiogenesis and enhancing vascular permeability [33]. Besides, E-cadherin, vimentin, and N-cadherin are all epithelial-mesenchymal transformation (EMT)-related proteins. EMT means that under certain conditions, epithelial cells lose their epithelial phenotypic characteristics and connections to each other, acquire stromal-like characteristics and motor ability, and can leave the in situ tissue [36]. EMT can enable stationary tumor cells to acquire motor ability, making tumor metastasis possible [37]. Furthermore, SDAD1 promotes the proliferation of COAD cells by reducing apoptosis [38]. Our results corroborated that the downregulated LINC01232 could promote p53, Bax and E-cadherin expressions, while suppressing Bcl-2, VEGF, vimentin, N-cadherin, and SDAD1 expressions, but such effects were reversed by miR-181a-5p inhibitor.

In summary, this study provides an overview of the role of LINC01232 in regulating COAD cell proliferation, migration, invasion, and angiogenesis. Further discussion revealed that the influence of LINC01232 on COAD progression is achieved by downregulating miR-181a-5p level. Our results may provide a potential therapeutic target for the treatment of COAD.

## Figures and Tables

**Table 1:** Specific primer sequences for quantitative reverse transcription polymerase chain reaction.

Gene	Primer sequence	Species
miR-181a-5p	5’-CTGTCGGTGAGTGTCGTAT-3’	Human
	5’-GTGCAATTGCCGACT-3’	
U6	5’-CTCGCTTCGGCAGCACA-3’	Human
	5’-AACGCTTCACGAATTTGCGT-3’	
LINC01232	5’-AGGATGCGCCTAAGAAAGGG-3’	Human
	5’-CCGGGGGATTGAGGAAACAT-3’	
p53	5’-GAGGTTGGCTCTGACTGTACC-3’	Human
	5’-TCCGTCCCAGTAGATTACCAC-3	
p16	5’-GGGTTTTCGTGGTTCACATCC-3’	Human
	5’-CTAGACGCTGGCTCCTCAGTA-3	
c-myc	5’-GTCAAGAGGCGAACACACAAC-3’	Human
	5’-TTGGACGGACAGGATGTATGC-3	
Bcl-2	5’-GGTGGGGTCATGTGTGTGG-3’	Human
	5’-CGGTTCAGGTACTCAGTCATCC-3	
CyclinD1	5’-CAATGACCCCGCACGATTTC-3’	Human
	5’-CATGGAGGGCGGATTGGAA-3	
β-actin	5’-CATGTACGTTGCTATCCAGGC-3’	Human
	5’-CTCCTTAATGTCACGCACGAT-3’	
